# Caliper measurements of femoral resection thickness in kinematic alignment total knee arthroplasty are accurate and reproducible

**DOI:** 10.1002/jeo2.70485

**Published:** 2025-11-03

**Authors:** Alexander J. Nedopil, Saahil Sandhar, Stephen M. Howell, Maury L. Hull

**Affiliations:** ^1^ Department of Orthopaedic Surgery König‐Ludwig‐Haus, University of Würzburg Würzburg Germany; ^2^ School of Medicine University of California Davis Sacramento California USA; ^3^ Department of Biomedical Engineering University of California Davis Davis California USA; ^4^ Department of Biomedical Engineering, Department of Mechanical Engineering, Department of Orthopaedic Surgery University of California Davis Davis California USA

**Keywords:** intraclass correlation coefficient, knee replacement, precision, random error, repeatability, systematic error

## Abstract

**Purpose:**

To resurface the patient's knee to the prearthritic state in caliper‐verified kinematic alignment (KA) total knee arthroplasty, the thickness of femoral resections after adjusting for saw blade kerf and wear should equal the femoral component. The primary objective was to determine accuracy of caliper measurement by quantifying the systematic (i.e., bias) and random (i.e., precision) errors of both unworn and worn articular surfaces from varus and valgus knees. The secondary objective was to assess reproducibility.

**Methods:**

In this diagnostic test study, thickness of each femoral resection from the first patients with 20 varus knees and the first patients with 20 valgus knees was measured by the surgeon using a caliper with 10 mm wide jaws. Subsequently, an optical method established the gold standard thickness. From the difference between caliper and optical measurements, the mean error (bias) and the standard deviation of the error (precision) were computed. Three observers measured resection thickness in three trials on both worn and unworn surfaces for 12 patients with varus knees. Repeatability and reproducibility were assessed by computing the variance components from a two‐factor analysis of variance and corresponding intraclass correlation coefficient (ICC) values.

**Results:**

For worn and unworn surfaces from both varus and valgus knees, the bias was negligible and the precision was less than or equal to the 0.5 mm resolution of the caliper. For worn and unworn surfaces from varus knees, ICC values were > 0.95 for both repeatability and reproducibility.

**Conclusions:**

The caliper used in this study is a sufficiently accurate tool for measuring resection thickness intraoperatively on worn and unworn femoral articular surfaces regardless of knee deformity. The error is less than the resolution of the caliper and the reproducibility is high thus giving surgeons confidence that the native limb and joint lines will be restored provided that the measured resection thickness after correcting for sawblade kerf and wear equals the femoral component thickness to within ±0.5 mm.

**Level of Evidence:**

N/A.

**Abbreviations**
ANOVA: analysis of variance
BMI: body mass index
F‐E: flexion‐extension
ICC: intraclass correlation coefficient
I‐E: internal‐external
KA: kinematic alignment
MA: mechanical alignment
TKA: total knee arthroplasty

## INTRODUCTION

Kinematic alignment (KA) total knee arthroplasty (TKA), which is performed without restrictions on preoperative deformity and/or flexion contracture, is a preferable alternative to mechanical alignment (MA) TKA. Since MA changes the native limb and joint line alignments in 98% of patients [9] whereas KA TKA restores native limb and joint line alignments, release of collateral ligaments is needed in the majority of MA cases [3, 12, 13, 14, 33] but release of these ligaments is avoided in KA [17, 26]. Patient‐reported outcomes scores for KA are generally superior to MA [5, 6, 7, 8, 10, 21, 23, 24, 34], biomechanical function is closely restored to native using components which retain the posterior cruciate ligament (PCL) and ensure that the axial rotation axis passes through the medial tibial compartment [11], and the risk of tibial baseplate loosening is minimal [17, 22, 27, 28, 29].

To restore native limb and joint line alignments, the femur must be resurfaced to the prearthritic state. Resurfacing requires that the thickness of each femoral resection matches the thickness of the femoral component after accounting for saw blade kerf and wear. To ensure that this requirement is met in practice, resection thickness at the apex should be measured intraoperatively and the thickness checked against the thickness of the femoral component. Where the articular surface is unworn, the resection thickness should equal that of the femoral component after compensating 1 mm for saw blade kerf. Where the articular surface is worn, a 2 mm compensation for cartilage thickness has been used so that resection thickness should equal femoral component thickness after the 2 mm compensation plus 1 mm for saw blade kerf [26]. Given the need to measure resection thickness intraoperatively, a validated method is needed. Although using a caliper to measure resection thickness is common practice [16, 26], the accuracy of this measurement has yet to be quantified.

To fully characterise measurement accuracy, two error quantities must be determined [1]. One is the systematic error (also known as bias or trueness) and the other is the random error (also known as precision or repeatability). In addition to the errors, it also is of interest to assess reproducibility (i.e., consistency in measurement between different operators). A thorough assessment of accuracy and reproducibility is necessary because judgement must be exercised in reading the caliper to the nearest 0.5 mm which is the practical resolution when reading the scale visually.

Considering the importance of knowing the accuracy to correctly restore native limb alignment and joint lines, the primary objective was to quantify both the bias and precision errors on worn and unworn articular surfaces using resections from varus and valgus knees. A secondary objective was to assess reproducibility. Our hypotheses were that bias would be negligible, that precision would be within the 0.5 mm resolution of the caliper, and that reproducibility would be excellent. If our hypotheses were supported, then surgeons could place confidence in using the caliper as a measurement tool to ensure that resection thickness matches that of the femoral component thereby restoring native limb and joint line alignments.

## MATERIALS AND METHODS

This diagnostic test study analysed resections from patients who underwent caliper‐verified KA TKA performed using manual instruments between 1 January 2025 and 31 March 2025. Each patient was treated regardless of the severity of varus or valgus deformity and the degree of flexion contracture. Included were those who had a KA‐optimised femoral component (GMK SpheriKA, Medacta International, www.medacta.com, accessed 11 March 2025). Individuals with a history of knee fractures treated with open reduction and internal fixation, inflammatory or septic arthritis, lower extremity neurologic disorders, no cartilage wear on either femoral condyle, or cartilage wear on both femoral condyles were excluded. Each patient fulfilled the Centers for Medicare and Medicaid Services guidelines for medical necessity for TKA and presented with Kellgren–Lawrence Grade III–IV osteoarthritis.

The surgical procedure was the same for all patients. After knee exposure, the surgeon inspected the distal femoral condyles and identified the unworn distal condyle (medial, lateral, both or none). The distal referencing guide was positioned. If the paddle of the distal referencing guide contacted partially worn cartilage, then the cartilage was removed to subchondral bone. A 2 mm shim was introduced to compensate for the worn and removed cartilage and the distal resections were performed. After exposure of the posterior condyles, identification, and removal of partially worn cartilage (if present) to the subchondral bone, the posterior resections were performed using a posterior referencing guide set at neutral rotation with a 2 mm shim to compensate for cartilage wear.

**Figure 1:**
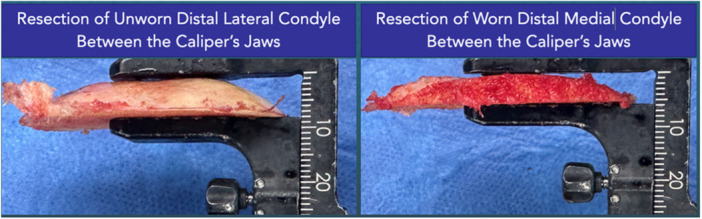
Photograph of caliper being used to measure thickness at the apex on unworn (left) and worn (right) distal femoral resections.

**Figure 2:**
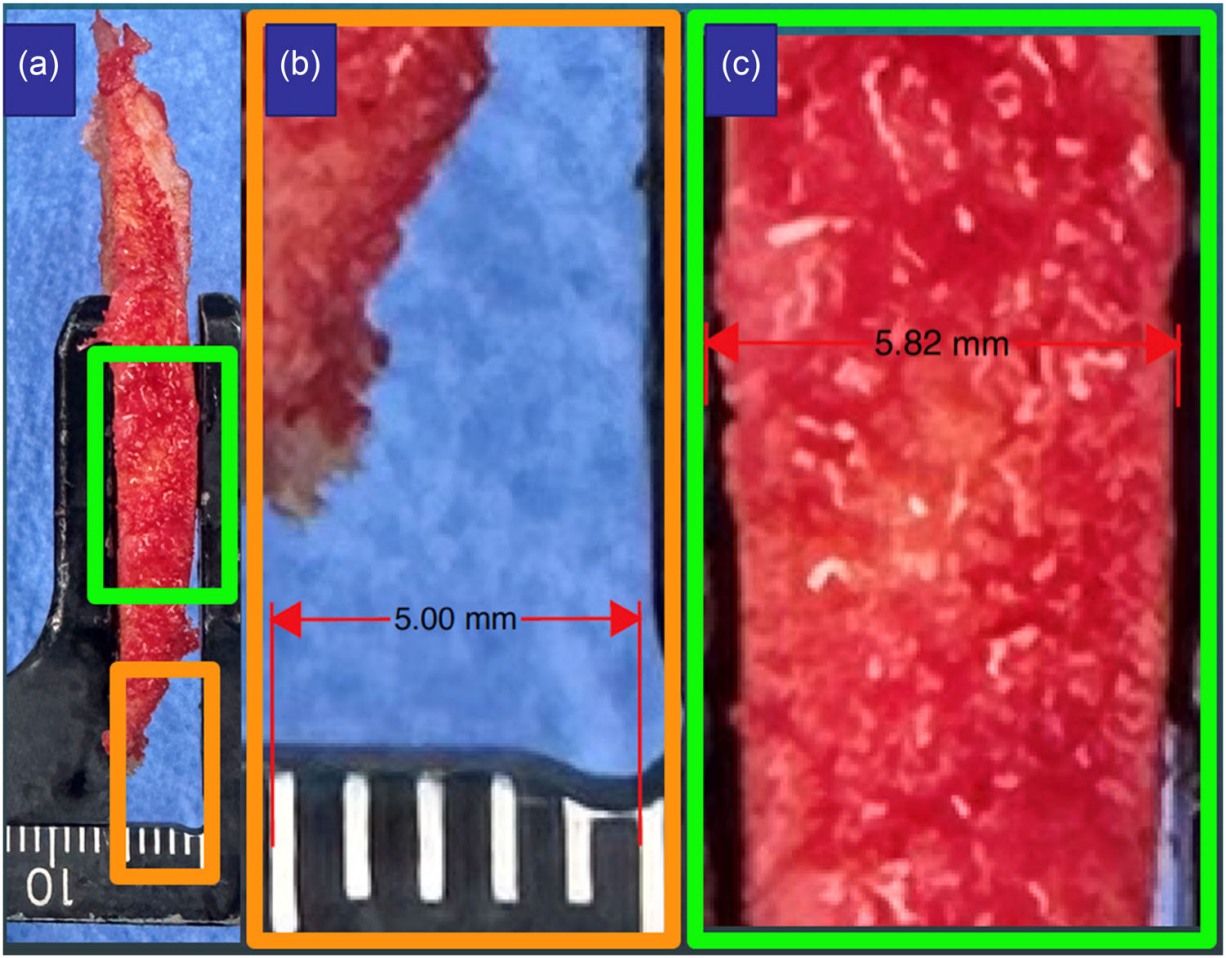
Composite illustrates the measurement of resection thickness at the apex using the Adobe Pro software. The resection was photographed (a). The image was calibrated to the scale on the caliper (b). The measurement tool in the software was used to determine the resection thickness (c).

For the 40 patients used for the first objective, the surgeon measured the thickness of each femoral resection at the apex with a caliper (Model 02.12.10.0728, Medacta International, Castel San Pietro, Switzerland) and recorded the results. The caliper featured jaws 10 mm wide thus ensuring that the measurement reflected maximum thickness. Following this measurement, the caliper was placed on each resection and the scale was photographed (Figure 1). The photograph was used to establish the gold standard for the resection thickness. The photograph was opened in Adobe Pro (Adobe Inc., San Jose, CA). The scale on the caliper served to calibrate the image using the software's calibration tool and the measurement tool was used to measure the thickness of the resection (Figure 2).

For the 12 patients used for the second objective, unworn and worn femoral resections from each patient (i.e. 24 resections total) were measured with the caliper three times by three observers. The observers were the surgeon, the physician's assistant, and the scrub technician. To ensure independent measurements by each observer, the measurements were made at three different times during the surgery. The times were after each resection, during assessment of the knee with trial components, and after implantation of the final components.

### Statistical analysis

To quantify the errors, the error of each caliper measurement was determined by subtracting the gold standard value from the caliper value. From these values, the bias was computed as the mean error and the precision was computed as the standard deviation of the error for each resection for varus and valgus knees. This yielded 8 pairs of error statistics (4 resections × 2 deformities). Ninety‐five percent confidence limits were placed on each pair of error statistics.

To assess reproducibility, a two‐factor analysis of variance (ANOVA) was performed with one factor at three levels for the observer and the other factor at 12 levels for the patients. The variance components for the observer, patients, and error were used to determine intraclass correlation coefficient (ICC) values for repeatability and reproducibility [4]. The standard deviation of the variance component for the observer quantified the variability introduced by the observer in relation to the variability introduced by the measurement method (i.e., intraobserver). ICC values > 0.90 indicate excellent agreement and 0.75–0.90 indicate good agreement.

## RESULTS

The analysis involved a total of 52 patients. The primary objective involved analysing results from the first 20 patients with varus knees and the first 20 patients with valgus knees (Table 1) whereas the secondary objective involved analysing results from the first 12 patients with varus knees (Table 2). Note that the patients involved with the secondary objective were different than those involved with the primary objective.

**Table 1:** Summary of demographics for the 40 patients used to quantify accuracy. Values are mean ± standard deviation.

Preoperative demographics and clinical characteristics	20 Patients with pre‐operative varus deformity	20 Patients with pre‐operative valgus deformity
Age	66 ± 10	68 ± 9
Sex	Males *N* = 13, females *N* = 7	Males *N* = 5, females *N* = 15
Body mass index (BMI) (kg/m²)	32 ± 6	32 ± 4
Knee Injury and Osteoarthritis Outcome Score	46 ± 12	46 ± 18
Oxford Knee Score	20 ± 9	22 ± 9
Knee extension (°)	7 ± 7	10 ± 5
Knee flexion (°)	114 ± 10	109 ± 9
Range of motion (°)	106 ± 14	99 ± 10
Radiographic anatomic tibiofemoral angle (+valgus/− varus) (°)	1 ± 3	9 ± 4

*Note*: Values are mean ± standard deviation.

**Table 2:** Summary of demographics for the 12 patients used to assess reproducibility. Values are mean ± standard deviation.

Preoperative demographics and clinical characteristics	12 Patients with pre‐operative varus deformity
Age	63 ± 10
Sex	Males *N* = 7, females *N* = 5
Body mass index (BMI) (kg/m²)	32 ± 7
Knee Injury and Osteoarthritis Outcome Score	45 ± 14
Oxford Knee Score	21 ± 9
Knee extension (°)	5 ± 4
Knee flexion (°)	115 ± 11
Range of motion (°)	110 ± 12
Radiographic anatomic tibiofemoral angle (+valgus) (°)	1 ± 4

For the 20 varus knees examined and the 20 valgus knees examined (Table 1), errors were comparable for worn and unworn articular surfaces (Table 3). Bias errors were less than or equal to than 0.2 mm and 95% confidence limits generally included 0 mm. Precision errors were ≤ 0.5 mm and the upper 95% confidence limits were ≤ 0.8 mm.

**Table 3:** Summary of accuracy results including 95% confidence limits in parentheses.

	Resection error (mm)			
	Distal medial	Distal lateral	Posterior medial	Posterior lateral
Varus knees				
Bias	−0.2 (−0.3 to −0.1)	0.0 (−0.1 to 0.1)	−0.1 (−0.3 to 0.0)	0.2 (0.1–0.3)
Precision	0.4 (0.3–0.6)	0.5 (0.4–0.7)	0.5 (0.4–0.8)	0.5 (0.4–0.8)
Valgus knees				
Bias	0.0 (−0.1 to 0.0)	0.0 (−0.1 to 0.1)	0.1 (0.1–0.2)	0.1 (0.0–0.2)
Precision	0.3 (0.3–0.5)	0.4 (0.3–0.5)	0.2 (0.2–0.3)	0.4 (0.3–0.6)

Based on the 12 varus knees examined (Table 2), ICC values were excellent. The ICC values for repeatability (i.e., intraobserver) and reproducibility (i.e. interobserver) were > 0.95. The intraobserver variability was 0.1 mm for both unworn and worn surfaces and the interobserver variability was 0.5 mm and 0.4 mm for unworn and worn surfaces, respectively.

## DISCUSSION

The most important findings were threefold. First, the only measurable error was the precision which was less than or equal to 0.5 mm. Second, the error was unaffected by worn versus unworn articular surfaces. Finally, ICC values for reproducibility were excellent.

To put the errors into proper perspective, the surgical alignment goal of KA must be considered. When conceived [18], the outcome goal of KA was to restore native knee function. Achieving this outcome goal is predicated on restoring the three kinematic axes of the knee joint to native [20]. Focusing on the tibiofemoral joint, the primary axis about which the tibia rotates is the flexion–extension (F–E) axis, which is fixed in the femur [2, 15, 30]. Accordingly, to restore this axis to native, one requirement is that the articular surfaces of the arthritic femur be restored to native (i.e., femur must be resurfaced to the prearthritic state). Hence, any material removed from the articular surfaces during surgery must be replaced by an equal amount by the femoral component.

To ensure that this requirement is satisfied intraoperatively, caliper‐verified KA TKA was developed [16]. This surgical technique relies on making caliper measurements of thickness at the apex of the resection and comparing the measured thickness to the corresponding thickness of the femoral component. After correcting for sawblade kerf and any articular surface wear, ideally the corrected measured thickness and the component thickness should be equal.

If the caliper‐measured thickness is prone to large error however, then this would lead to femoral component misalignment and possible complications in not only the tibiofemoral joint but also the patellofemoral joint since the posterior resections set the internal–external (I–E) rotation of the femoral component which in turn affects patellar tracking. For example, if the femoral component is malrotated internally, then this rotation would medialize the patella increasing risk of patellar instability. By limiting the precision to the resolution of the caliper, our results show that the caliper design used in this study is an effective measurement tool for comparing resection thickness to the corresponding thickness of the femoral component.

Since caliper designs differ, the ability of different designs to achieve comparable accuracy to that herein merits consideration. The caliper design used in this study offered a jaw width of 10 mm. Since an unworn cartilage surface is dome shaped, the jaw width ensured that the surface of the caliper contacted the apex of the dome so that the measurement indicated the maximum thickness of the resection (Figure 1). Using caliper designs with narrower jaws could introduce error if the caliper jaw surface did not rest on the apex of the dome which would require properly locating the caliper jaw in both the anterior–posterior and medial–lateral directions. Another source of error is failure to measure thickness perpendicular to the resection plane which would occur if the caliper was angled relative to the resected surface. To confirm the importance of these sources of error, the hypothesis that a narrow‐jaw caliper has significantly greater error than a wide‐jaw caliper would need to be tested.

Since the caliper is used to measure thickness of both worn and unworn surfaces, an independent variable of this study was the surface condition. Since unworn surfaces have a smooth surface whereas worn surfaces required cartilage removal to the subchondral bone resulting in a rough surface, the surface condition could possibly have affected the error. However, our results showed that the error was unaffected by the surface condition (Table 3).

To assess reproducibility, both the ICC value for reproducibility and the actual variability introduced by the observer were quantified. The reason for using two quantities is traced to the limitations in relying solely on the ICC value. To appreciate the limitation in using solely the ICC value, the magnitudes of the respective variance terms should be recognised [31]. If the between subject variance is large relative to the between observer variance, then the ICC for reproducibility will be high. To illustrate, in our case for the unworn resections, variance terms for between subject, between observer, and error were 9.71, 0.21 and 0.05, respectively. With these values, the ICC for reproducibility becomes 9.71/(9.71 + 0.21 + 0.05) = 0.97 which is exceedingly high whereas the actual error introduced by the observer was 0.5 mm (i.e., square root of corresponding variance). Accordingly, the ICC alone has limited usefulness in indicating reproducibility. Nevertheless, like the precision, the error introduced by the observer was limited to the resolution of the caliper.

Since accuracy of different types of instrumentation in making resections is a topic of high interest, it is useful to consider our results in this context. A comparison of resection accuracy for caliper‐verified KA using manual instruments versus robotic arm assisted methods and patient‐specific instruments has been made in detail in a recently published paper [32]. In this comparison, the resection accuracy of manual instruments was markedly better than that of robotic arm assisted systems and patient‐specific instruments. However, to determine resection accuracy, resection thickness was measured with a caliper in some studies. A general limitation not recognised in any study determining resection accuracy is that any inaccuracy (i.e., error) stems from two sources: (1) the caliper and (2) the instrumentation. Hence, an implicit assumption was that the caliper introduced negligible error which our study shows is not the case for precision. As a result, accuracy of the instrumentation considering precision was confounded. Furthermore, caliper accuracy may be considerably worse than that reported herein if narrow‐jaw calipers were used as noted above. To correctly determine instrumentation accuracy as distinct from caliper accuracy, accuracy due to the particular caliper used must be determined and resection accuracy corrected to reflect caliper error.

The main limitation to this study was that two non‐surgeons served as two of the three observers for the reproducibility study. Accordingly, a concern might be that the precision of their measurements was inflated above that of the surgeon with extensive experience measuring resection thickness. However, notwithstanding minimal training which consisted of instructing them to measure at the thickest point, their precisions were either the same or less than that of the surgeon. Hence, the caliper measurement is robust in that it does not depend on observer experience.

Another possible limitation surrounds the method of gold‐standard measurement. Since the optical measurement method used for the gold standard has a resolution of 0.01 mm whereas the caliper measurement has a resolution of 0.5 mm, the optical method was deemed a viable gold standard. Although repeatability and reproducibility of the gold standard method were not determined, this would not have affected our results given that the resolution of the gold standard was 50 times better than the caliper.

A final possible limitation concerns the experience level of the surgeon. While an experienced surgeon performed the KA TKAs, it is unlikely that surgeon experience would affect our results. This is because the accuracy in making the femoral resections is the same for experienced and inexperienced surgeons [19, 25].

## CONCLUSION

The caliper with 10 mm wide jaws is a sufficiently accurate tool for measuring resection thickness intraoperatively on worn and unworn articular surfaces regardless of knee deformity. The precision is generally comparable to the resolution of the caliper as is the interobserver error thus giving surgeons confidence that the native limb and joint lines will be restored provided that the measured resection thickness equals the femoral component thickness within ± 0.5 mm.

## AUTHOR CONTRIBUTIONS

Alexander J. Nedopil conceived the study, developed the gold standard method to measure cartilage thickness, and gathered thickness data measured with the caliper. Saahil Sandhar determined cartilage thickness from photographs; Stephen M. Howell edited the manuscript. Maury L. Hull wrote and revised the manuscript.

## CONFLICT OF INTEREST STATEMENT

Alexander J. Nedopil is a paid consultant and receives royalties from Medacta. Stephen M. Howell is a paid consultant, a paid speaker, and receives royalties from Medacta. Maury L. Hull is a paid speaker and receives research funding from Medacta.

## ETHICS STATEMENT

None declared.

## Data Availability

Date are available upon request.
